# An Observational Study of Lung Function Alterations in Stone Quarry Workers of North Karnataka

**DOI:** 10.7759/cureus.80838

**Published:** 2025-03-19

**Authors:** Sharanagouda M Patil, Manjunatha Aithala, Praveen Ganganahalli

**Affiliations:** 1 Physiology, Bijapur Liberal District Education (BLDE) (Deemed to be University) Shri B. M. Patil Medical College, Hospital and Research Center, Vijayapura, IND; 2 Physiology, Al-Ameen Medical College, Bijapur, IND; 3 Community Medicine, Bijapur Liberal District Education (BLDE) (Deemed to be University) Shri B. M. Patil Medical College, Hospital and Research Center, Vijayapura, IND

**Keywords:** fev1, fvc, lung function tests, mep, pefr, stone quarry workers

## Abstract

Introduction

The disorganized stone quarrying industry creates much noise and pollution for valuable commodities such as coal, metal, and stones throughout the extraction process. It produces dust particles with varying aerodynamic sizes between 1 and 100 microns composed of silica, organic solvents, and heavy metals. The objective of this study is to determine the effects of stone dust on pulmonary functions and their correlations with sociodemographic variables among quarry stone workers.

Methodology

The study group consisted of 50 male subjects working in a quarry exposed to stone dust, and the control group comprised 50 matched male subjects who were hospital employees. Detailed anthropometric and physiological data and pulmonary functions were recorded using computerized Spiro Excel. The parameters recorded were forced vital capacity (FVC) in mL, forced expiratory volume in the first second (FEV1) in mL, the percentage of forced expiratory volume in one second (FEV1%), and maximum expiratory pressure (MEP) in mmHg recorded using the modified Blacks apparatus.

Observation

There was a significant reduction in peak expiratory flow rate (PEFR) (p=0.004) in the study group (138.4±62.80 L/minute) as compared to the control group (272.8±327.6 L/minute). Also, there was a significant reduction in MEP (p=0.16) in the study group (23.6±12.4 mmHg) as compared to the control group (38.4±227 mmHg). There was no significant decrease in other parameters in the study group compared to the controls.

Conclusion

Lung function parameters were negatively correlated with the duration of exposure. We attribute this reduction in lung function tests to respiratory muscle weakness. Therefore, breathing exercises must be available to such workers to strengthen the respiratory muscles and improve lung function tests.

## Introduction

The disorganized stone quarrying industry creates much noise and pollution for valuable commodities such as coal, metal, and stones throughout the extraction process. Drilling, blasting, and crushing are steps in the quarrying process that pollute the environment by releasing dust. Although the dust produced impacts nearby residents, there is a greater need for these aggregates because they are necessary building materials. The largest source of emissions is quarries. It makes dust particles with varying aerodynamic sizes between 1 and 100 microns composed of silica, organic solvents, and heavy metals [1,2].

It has been demonstrated that people who live near mining sites and breathe in dust from those operations are more likely to develop lung tumors and other respiratory ailments. Like other mining operations, stone quarry operations typically release inhalable particles of various sizes into the air, which nearby residents and employees may quickly inhale. Asthma, lung tumors, pneumoconiosis, and deterioration in respiratory health are just some of the ways that jeopardize the health of employees. It is well known that silica particles contribute to oxidative stress as a mechanism of toxicity. Quarry stones, commonly used in construction and building projects, are a primary source of these particles [3,4].

This frequently has several negative health effects. Mining dust has historically been linked to several deadly diseases, including black lung, which results from an inflammatory reaction in the lungs and a subsequent deterioration in lung function, and pneumoconiosis, which is caused by silicosis or the common coal worker pneumoconiosis. Inhalable quarry stone dusts remarkably affect the lungs because they can cause congestive blockage due to an inflammatory response and the propensity for lung-related disorders to worsen over time. Quarrying work is often characterized by hazardous and challenging work conditions that involve manual handling of material, lifting of heavy objects, movements and tasks that are recurrent, manual exertion of forces, and possible exposure to segmental or whole-body vibration [5-7].

In light of this, a comparative observational study was designed to determine the effects of stone dust on pulmonary functions in quarry stone workers and non-quarry workers, as well as the relationships between these effects and variables.

## Materials and methods

This comparative cross-sectional study was conducted among quarry workers exposed to stone dust compared with the general population of the northern region of Karnataka who were not exposed to it. The duration of data collection was from August 2013 to January 2014 using the convenience sampling technique and structured proforma.

Sample size

To determine the functional modification among quarry workers, a sample size of 100 was determined using the following formula: n=4pq/d2, considering that the proportion of the bad outcome among persons exposed to stone dust was 50%. There were 100 participants in total, 50 in each of the research and control groups. The control group's subjects were chosen to match the study group's age and sex.

Inclusion criteria

The study group comprised 50 unskilled laborers exposed to stone dust for more than five years. The survey only included employees who appeared to be in good health. A comprehensive clinical examination and history collection determined each subject's health status. The control group comprised 50 employees who worked at the current institution and were not exposed to dust. The control group's subjects were chosen to match the study group's age and sex.

Exclusion criteria

Participants with a history of congenital malformations, endocrine diseases, cardiac disorders, or smoking were not allowed to participate in the study.

Study tools

Computerized Spiro Excel recorded detailed anthropometric, physiological, and pulmonary data. The modified Blacks apparatus was used to record the following parameters: maximum expiratory pressure (MEP), forced expiratory volume in one second (FEV1), the percentage of forced expiratory volume in one second (FEV1%), and forced vital capacity (FVC).

Physical anthropometry of subjects

Each subject's height (in cm) was measured while standing barefoot to within 0.1 cm. Weight (in km) was measured using a standardized machine with the least amount of clothing closest to 0.1 kg. Using a standardized tailor tape, the chest circumference was measured in the deep inspiration posture, at the nipple level, and with the least amount of clothing possible, to within 0.1 cm. Each subject's body mass index (BMI) (in kg/m^2^) was determined using the Quetelet index, as well as their height in m^2^ and weight in kg.

Physiological parameters

The right radial artery was palpated to determine the pulse rate in bpm. A mercury sphygmomanometer was used to measure the systolic and diastolic blood pressure in mmHg. The formula DBP±1/3 pulse pressure (PP) was used to calculate the mean arterial pressure (MAP), which was expressed in mmHg. The respiratory rate was expressed in cycles per minute using a manual method.

Pulmonary function parameters

Spiropac (MEDICAID) was used to record each subject's pulmonary function parameters, including forced vital capacity (FVC) in mL, forced expiratory volume at the first second (FEV1) in mL, and the percentage of forced expiratory volume in one second (FEV1%), which was computed mathematically using the following formula: FEV1% is equal to FEV1/FVC×100, peak expiratory flow rate (PEFR) in L/min using a mini-Wright peak flow meter, and maximum expiratory pressure (MEP) in mmHg using the modified Blacks apparatus.

Statistical analysis

A statistician was consulted while analyzing the collected data for regression, correlation, mean, standard deviation, and unpaired Student's t-test. The association between the pulmonary function tests (PFTs) of the stone quarry workers and the variables related to anthropometry, work history, and demography was examined using Pearson's correlation. The workers' PFTs were also contrasted with those of healthy participants.

## Results

A total of 100 participants were enrolled, according to the inclusion and exclusion criteria, with 50 participants each in the study and control groups.

The study group's mean age was 40 years, while the control group was 38 years, as shown in Table 1. This difference was not found to be statistically significant. Similarly, the participants' anthropometric measurements did not significantly differ between the two groups, suggesting that the two groups were matched to prevent bias. Both group members' body mass index (BMI) were within the normal range.

**Table 1:** Anthropometric measurements of the participants (N=100) BMI: body mass index

Parameters	Study group (n=50)	Control group (n=50)	Unpaired t-test (p-value)
Age (years)	40.12±12.66	38.44±11.32	0.6995 (0.485)
Height (cm)	161.1±8.07	161.1±7.27	0.000 (0.999)
Weight (cm)	59.8±13.9	58.42±11.1	0.548 (0.584)
BMI (kg/m^2^)	22.86±5.04	21.8±4.87	1.069 (0.287)
Hip circumference (cm)	89.24±8.9	87.04±1.36	1.728 (0.08)
Waist circumference (cm)	85.6±9.44	82.6±9.42	1.591 (0.114)

According to Table 2, the physiological parameters of the members of the two groups were almost identical, and any discrepancies were not deemed statistically significant. Upon inspection, the participants in the study and control groups had a mean systolic blood pressure of 130 and 124 mmHg, respectively, and diastolic blood pressure of 80 and 78 mmHg, respectively, indicating that they were normotensives.

**Table 2:** Physiological parameters of the study group versus the control group (N=100) BP: blood pressure

Parameters	Study group (n=50)	Control group (n=50)	Unpaired t-test (p-value)
Pulse rate (beats/minute)	77±8	74±8	1.803 (0.07)
Respiratory rate (cycles/minute)	19±2	18±2	1.331 (0.186)
Systolic BP (mmHg)	130±18	124±20	0.4255 (0.671)
Diastolic BP (mmHg)	80±10	78±10	0.626 (0.532)

Table 3 shows that forced vital capacity (FVC) and forced expiratory volume in one second (FVC1) were lower in the study group compared to the control group, which was found to be statistically significant. The percentage of forced expiratory volume in one second (FEV1%) was nearly equal in both groups. While the study group's peak expiratory flow rate (PEFR) was significantly lower (138.4 L/minute) than that of the control group (272.8 L/minute), the study group's maximum expiratory pressure (MEP) was also significantly lower (23.6 mmHg) than that of the control group (32.4 mmHg).

**Table 3:** Pulmonary parameters of the study group versus the control group (N=100) FVC: forced vital capacity, FEV1: forced expiratory volume in one second, FEV1%: percentage of forced expiratory volume in one second, PEFR: peak expiratory flow rate, MEP: maximum expiratory pressure

Parameters	Study group (n=50)	Control group (n=50)	Unpaired t-test (p-value)
FVC (L)	3.03±0.95	3.5±0.90	2.540 (0.012)
FEV1 (L)	1.63±0.50	3.64±1.44	9.324 (0.0001)
FEV1%	85.04±19.4	86.22±23.12	0.276 (0.782)
PEFR (L/minute)	138.4±62.80	272.8±327.6	2.849 (0.005)
MEP (mmHg)	23.6±12.4	32.4±22.7	2.406 (0.01)

## Discussion

There was a significant reduction in PEFR (p=0.005) in the study group (138.4±62.80) as compared to the control group (272.8±327.6). There was also a substantial reduction in MEP (p=0.01) in the study group (23.6±12.4) as compared to the control group (38.4±227). There was no significant decrease in other parameters in the study group compared to the control group.

According to Arumugam et al., who included 670 participants with a median age of 37 years, quarry workers with more airflow obstruction and infections such as tuberculosis had considerably lower mean PFT measures. Smoking and working in a quarry for longer increased the risk of airflow obstruction among quarry workers [8].

According to Solanki et al., the average age of stone cutters was 31.43±9.18 years, and their average length of time on the job was 12.32±6.11. Overall, 31.61% of individuals experienced wheezing, 10.29% complained of chest pain, and 50.24% complained of a persistent cough. Data analysis revealed a substantial difference in FEV1, FVC, and FEV1/FVC following three months of physiotherapeutic intervention [9].

Hamzah et al. found that the proportion of the percentage of forced expiratory volume in one second per forced vital capacity (FEV1%/FVC) was significantly lower (t=-3.729, p<0.0001) than in the comparison group. Smoking was shown to be substantially associated with FVC (adjusted b=-0.41, p=0.024), but age had a negative correlation with both FEV1 (adjusted b=-0.03, p=0.003) and FEV1%/FVC (adjusted b=-0.42, p=0.004) [10].

The study by Rajanayagam et al. shows that obstructive or restrictive lung disorders increased with continuous dust exposure and length of work in the quarry. The test subject's mean forced expiratory volume (FEV) (2.30 L versus 3.19 L) (p=0.001) and forced vital capacity (FVC) (3.05 L versus 3.59 L) (p=0.001) were significantly decreased, according to the data. Simple linear regression analysis revealed that only an increase in the duration of silica dust exposure was significantly associated with a reduction in pulmonary function tests, specifically in FVC (p=0.019), FEV_3_ (p=0.016), FEF_25_ (p=0.016), FEF_0.2-1.2_ (p=0.048), PEFR (p=0.019), and maximal voluntary ventilation (MVV) (p=0.001) values [1].

Sheikh et al. compared manual workers to non-manual workers and smokers to non-smokers, finding a statistically significant (p<0.05) decrease in lung function test values. Only an increase in the duration of silica dust exposure was found to be significantly associated with a reduction in pulmonary function tests, specifically in FVC (p=0.019), FEV_3_ (p=0.016), FEF_25_ (p=0.016), FEF_0.2-1.2_ (p=0.048), PEFR (p=0.019), and MVV (p=0.001) values, according to simple linear regression analysis [11].

Priya et al. concluded in their study that 33 (26.4%) study participants experienced dyspnea, 25 (20%) coughed, 17 (13.6%) produced sputum, 12 (9.6%) wheezed, four (3.2%) experienced chest pain, and four (3.2%) experienced hemoptysis. The forced expiratory volume in one second/forced vital capacity ratio was significantly inversely correlated with the stone sculptors' age, years of smoking, employment, comorbidities, and respiratory symptoms [12].

Silica dust had long-term effects on the pulmonary function parameters of stone quarry workers, according to the findings of other authors who have also studied these workers' pulmonary functions. Silica dust deteriorates pulmonary health [13]. Consideration must be given to a comfortable working environment, adequate ventilation, and suitable masks and eyewear supply for each employee. Awareness of quarry workers is necessary for improved health [14,15]. 

Strength of the study

The present study findings help us screen the workers for regular pulmonary function tests and early diagnosis and prevention of the compromise of lung functions.

Limitations of the study

The study was conducted in particular geographic areas and cannot be generalized to the entire population. Multicenter studies are needed.

## Conclusions

In this study on stone quarry workers, pulmonary function measures such as FVC, FEV1, FEV1%, PEFR, and MEP were lower in the study group than in the control group. This might lead to airway occlusion and weakening of the respiratory muscles, depending on the physical and chemical composition of the stone particles. As a result, it is recommended that individuals who are exposed to dust have regular recordings of simple, non-invasive, and dynamic pulmonary function tests. In clinical practice, this could help determine prognostication. Breathing exercises can improve lung function and strengthen the respiratory muscles.

## Appendices

The proforma for data collection is shown in Table 4.

**Table 4:** Proforma for data collection BMI: body mass index, FVC: forced vital capacity, FEV1: forced expiratory volume in one second, FEV1%: percentage of forced expiratory volume, PEFR: peak expiratory flow rate, MEP: maximum expiratory pressure

Age (in completed years)	
Gender	Male/female
Duration of work (in years)	
Height (cm)	
Weight (kg)	
BMI (kg/m^2^)	
Waist circumference (cm)	
Hip circumference (cm)	
Pulse rate (beats/minute)	
Respiratory rate (cycles/minute)	
Systolic blood pressure (mmHg)	
Diastolic blood pressure (mmHg)	
Pulmonary function tests	
FVC (L)	
FEV1 (L)	
FEV1%	
PEFR (L/minute)	
MEP (mmHg)	
